# E-Cadherin-Deficient Epithelial Cells Are Sensitive to HDAC Inhibitors

**DOI:** 10.3390/cancers14010175

**Published:** 2021-12-30

**Authors:** Lyvianne Decourtye-Espiard, Nicola Bougen-Zhukov, Tanis Godwin, Tom Brew, Emily Schulpen, Michael A. Black, Parry Guilford

**Affiliations:** Cancer Genetics Laboratory, Centre for Translational Cancer Research (Te Aho Matatū), Department of Biochemistry, University of Otago, Dunedin 9016, New Zealand; lyvianne.decourtye@otago.ac.nz (L.D.-E.); nicola.bougen-zhukov@otago.ac.nz (N.B.-Z.); tanis.godwin@otago.ac.nz (T.G.); tom.p.brew@gmail.com (T.B.); emily.schulpen@otago.ac.nz (E.S.); mik.black@otago.ac.nz (M.A.B.)

**Keywords:** HDGC, *CDH1*, E-cadherin, HDAC inhibitors, synthetic lethality

## Abstract

**Simple Summary:**

Inactivating mutations in the *CDH1* gene cause the cancer syndrome hereditary diffuse gastric cancer and are also frequent in sporadic diffuse gastric and lobular breast cancers. This research aimed to test whether cancers with *CDH1* mutations have heightened sensitivity to histone deacetylase (HDAC) inhibitors. The impact of these drugs was tested on several gastric and breast preclinical models with and without *CDH1* mutations. One HDAC inhibitor, entinostat, showed a strong inhibitory effect on each of the models with *CDH1* mutations. This study highlighted the potential beneficial impact of entinostat for the chemoprevention and/or treatment of gastric and breast cancers carrying *CDH1* mutations.

**Abstract:**

Inactivating germline mutations in the *CDH1* gene (encoding the E-cadherin protein) are the genetic hallmark of hereditary diffuse gastric cancer (HDGC), and somatic *CDH1* mutations are an early event in the development of sporadic diffuse gastric cancer (DGC) and lobular breast cancer (LBC). In this study, histone deacetylase (HDAC) inhibitors were tested for their ability to preferentially inhibit the growth of human cell lines (MCF10A and NCI-N87) and murine organoids lacking *CDH1* expression. *CDH1^−/−^* breast and gastric cells were more sensitive to the pan-HDAC inhibitors entinostat, pracinostat, mocetinostat and vorinostat than wild-type cells, with an elevated growth inhibition that was, in part, attributable to increased apoptosis. *CDH1*-null cells were also sensitive to more class-specific HDAC inhibitors, but compared to the pan-inhibitors, these effects were less robust to genetic background. Increased sensitivity to entinostat was also observed in gastric organoids with both *Cdh1* and *Tp53* deletions. However, the deletion of *Tp53* largely abrogated the sensitivity of the *Cdh1*-null organoids to pracinostat and mocetinostat. Finally, entinostat enhanced *Cdh1* expression in heterozygous *Cdh1^+/−^* murine organoids. In conclusion, entinostat is a promising drug for the chemoprevention and/or treatment of HDGC and may also be beneficial for the treatment of sporadic *CDH1*-deficient cancers.

## 1. Introduction

Hereditary diffuse gastric cancer (HDGC) is an autosomal dominant cancer syndrome defined by germline *CDH1* or, occasionally, *CTNNA1* variants in either an isolated individual with diffuse gastric cancer (DGC), or in a family with one or more cases [1]. Pathogenic germline *CDH1* mutations lead to a lifetime risk of developing DGC and lobular breast cancer (LBC) of up to 70% and 40%, respectively [1]. Tumor formation in HDGC requires inactivation of the retained wild-type *CDH1* allele by mechanisms including mutation and promoter hypermethylation [2]. The *CDH1* gene encodes E-cadherin, a homophilic cell-to-cell adhesion protein involved in the maintenance of cellular polarity, epithelial tissue architecture and tension sensing [3]. The loss of expression of E-cadherin has been associated with an increased ability of cells to detach, evade apoptotic machinery and acquire invasive and metastatic characteristics [4,5].

Early stage HDGC is characterized by relatively indolent, multifocal signet-ring cell carcinomas (SRCC) that are initially confined to the *lamina propria* (stage T1a) [6]. However, the prognosis worsens once these SRCC have invaded into the gastric submucosa. As a consequence, *CDH1* variants carriers from families with confirmed cases of DGC are advised to undergo a prophylactic total gastrectomy between 20 and 30 years of age. For carriers who do not pursue a gastrectomy, yearly endoscopy is recommended [1,7]. Following gastrectomy, long-term sequelae including nutritional and psychological effects need to be monitored. It is recommended that the risk of LBC is managed with annual MRI surveillance beginning at 30 years of age [1]. Given these risks and morbidities, it is clear that the quality of life of *CDH1* mutation carriers would benefit from the development of drugs that prevent or delay disease development [8].

Synthetic lethal (SL) approaches have previously been used to identify vulnerabilities in cells lacking *CDH1* expression [9,10]. Synthetic lethality is classically defined as a genetic interaction in which the loss of function of two genes at the same time leads to cell death, whereas the inactivation of either gene alone has little to no effect on cell viability. In HDGC therapy, we use the term “synthetic lethality” to refer to pharmacological inhibition that preferentially inhibits the growth or survival of cancer cells that lack E-cadherin expression relative to wild-type cells [11]. Previous genome-wide siRNA and drug screening studies have identified synthetic lethal vulnerabilities in MCF10A breast cells deficient for E-cadherin compared to wild-type MCF10A cells [12]. Of those SL vulnerabilities, multiple histone deacetylase (HDAC) inhibitors were identified. Moreover, dysregulation in HDAC expression has been linked to gastric cancer [13,14,15]. These studies support the potential efficacy of histone deacetylase inhibitors for the chemoprevention or treatment in HDGC.

Histone deacetylases are proteins involved in post-translational modification of diverse histone and non-histone proteins through the removal of acetyl groups from the ε-amino group of lysine [16,17]. HDACs are implicated in diverse biological processes, including tissue developmental programming and apoptosis, and have a well-established role in transcriptional control [18,19]. They are separated into four classes depending on their homology to yeast HDAC. Class I, II and IV HDACs are zinc-dependent metalloproteins, and Class III HDACs are NAD+-dependent [20]. Notably, Class II HDACs have low intrinsic enzymatic activity, but they participate in deacetylase activity through their engagement with the HDAC3-containing SMRT/NCoR repressive complex [21].

Dysregulation of the transcription of HDAC genes has been associated with numerous diseases, including neurodegenerative disorders such as Parkinson’s disease and Alzheimer’s disease, cardiac defects, obesity, diabetes, chronic obstructive pulmonary disease and cancer [22]. Overexpression of specific HDACs is observed in multiple malignancies, including gastric, colon, prostate and breast cancers [23]. In in vitro and in vivo preclinical cancer models, the inhibition of HDAC activity leads to terminal differentiation, cell-cycle arrest, autophagy and/or apoptosis [24,25,26,27]. As a result, several HDAC inhibitors are currently being tested in clinical trials, increasingly as part of combination therapy [20,22]. In this study, we focused on the effect of four pan-HDAC inhibitors, namely the benzamide derivatives entinostat and mocetinostat [28] and two hydroxamic acid derivatives, pracinostat and vorinostat [23]. Using 2D and 3D preclinical models with distinct genetic backgrounds, we demonstrate the potential utility of HDAC inhibitors for the chemoprevention of HDGC and treatment of sporadic E-cadherin-deficient cancers.

## 2. Materials and Methods

### 2.1. RNA-Seq

The correlation between HDACs and *CDH1* expression levels was assessed by using RNA-Seq data from the Stomach Adenocarcinoma (STAD) cohort in The Cancer Genome Atlas (TCGA), as previously described [9].

### 2.2. Cell Culture

MCF10A, a non-tumorigenic breast cell line (ATCC, CRL-10317) and MCF10A *CDH1^−/−^* cells (CLLS1042, Sigma-Aldrich, St Louis, MO, USA) were cultured in DMEM/F12/Glutamax (10565042, Thermo Fisher Scientific, Waltham, MA, USA) with 5% horse serum (16050, Thermo Fisher Scientific, Waltham, MA, USA), 10μg/μL neutral insulin (797139, Dunedin hospital, Dunedin, New Zealand), 500 ng/mL hydrocortisone (H0888, Sigma-Aldrich, St Louis, MO, USA), 100 ng/mL cholera toxin (C8052, Sigma-Aldrich, St Louis, MO, USA) and 20 ng/mL human epidermal growth factor (E9644, Sigma-Aldrich, St Louis, MO, USA). A *CDH1*-null strain of the gastric cancer cell line NCI-N87 (ATCC, CRL-5822) was generated using CRISPR-Cas9 [9]. Briefly, the sequence 5′-ATTCACATCCAGCACATCCA-3′ was used to target exon 10 of *CDH1* and induce a single base frameshift, followed by a stop codon. Isogenic NCI-N87 cells were cultured in DMEM/F12/Glutamax with 10% fetal bovine serum (10091148, Thermo Fisher Scientific, Waltham, MA, USA). Both cell lines were grown at 37 °C with 5% CO_2_.

### 2.3. Drugging Assay on Cell Culture

For drugging experiments, cells were seeded in a 96-well plate at 4000 or 10,000 cells/well for MCF10A and NCI-N87, respectively, for 48 h drugging (2500 and 6500 cells for 72 h experiment). On day one, nuclei on the outer well were counted by using 1 μg/mL Hoechst (H1399, Thermo Fisher Scientific, Waltham, MA, USA) on a Cytation imager (Biotek, Winooski, VT, USA), with the associated Gen5 software (Biotek, Winooski, VT, USA). If the ratio of WT vs. *CDH1^−/−^* cells was between 0.6 and 1.4, cells were processed for drugging. The different drugs used (Table S1) were solubilized in DMSO and diluted in the appropriate media. Only DMSO was added to the control wells to a concentration of 0.1 to 0.4% to match the concentration in the drugging wells. After 48 or 72 h, cells were stained by using 0.25% PFA, 0.075% Saponin and 1 μg/mL Hoechst in 1× PBS. Cell count was assessed by using Cytation 5 imager and normalized to the DMSO controls. Drugging was performed as technical triplicates for every concentration and combined.

### 2.4. Organoid Culture

Organoids were made by using inducible Cre-lox mice [29] that were crossed to obtain the desired genotype. The *CD44*-Cre/tdTomato (referred to as WT in this manuscript) express an inducible Cre recombinase under the control of the *CD44* promoter (Ozgene, Perth, Australia) and loxP-flanked STOP cassette, preventing the red fluorescent protein tdTomato (B6.Cg-Gt(ROSA)26Sortm9(CAG-tdTomato0Hze/J, The Jackson Laboratory, Bar Harbor, ME, USA). These organoids expressed robust tdTomato fluorescence following Cre induction. *Cdh1* deletion was generated by using *CD44*-Cre/*CDH1*^(fl/fl)^/tdTomato stomachs or mammary tissues (hereafter referred to as *Cdh1^−/−^*) from mice that possess loxP sites flanking exons 6 to 10 of *Cdh1* (B6.129-CDH1^tm2kom^/J, strain 005319, The Jackson Laboratory, Bar Harbor, ME, USA). Organoids with decreased *Cdh1* expression (*Cdh1^+/−^* organoids) were generated by using stomachs with only loxP sites on one allele of *Cdh1*. *Tp53* and *Cdh1* deletion were generated by using CD44-Cre/*Cdh1*^(fl/fl)^/*Tp53^(fl/fl)^*/tdTomato stomachs (named *Cdh1^−/−^/Tp53^−/−^* in this manuscript) with *Tp53* exons 2–10 flanked by loxP sites (B6.129P2-Tp53^tm1Brn^/J, strain 008462, The Jackson Laboratory, Bar Harbor, ME, USA). The antral tissue of the stomach and mammary gland was extracted from mice aged 6 to 8 weeks and washed in PBS and 100 µg/mL primocin (ant-pm-1, Invitrogen, Carlsbad, CA, USA). Stomachs were incubated in a chelation buffer made of 25 mM EDTA and 100 µg/mL primocin in PBS, and the gastric glands were filtered through a 70 µm cell strainer and resuspended in Matrigel (FAL356231, In Vitro Technologies, Mt Wellington, Auckland, New Zealand). Mammary tissues were incubated for 30 min in a collagenase solution (containing 75 mg collagenase A (17104019 Thermo Fisher Scientific, Waltham, MA, USA) and 150 mg Dispase (7105041, Thermo Fisher Scientific, Waltham, MA, USA) [30]. Mammary glands were then thoroughly washed in DMEM/F12 with primocin and resuspended in Matrigel.

Organoids were cultured in organoid complete media (Tables S2 and S3). Gastric organoids were passaged as single cells every 5–6 days and seeded at a concentration of 20 cells/µL of Matrigel. Mammary organoids were passaged every 7 days and seeded at a concentration of 60 cells/µL of Matrigel. Organoids were grown at 37 °C with 5% CO_2_.

### 2.5. Drugging Assay on Organoids

For drugging experiments, 250 (gastric) or 300 (mammary) cells/well were seeded in a 384-well plate (day 0). Organoids were induced with 0.5 µg/µL of endoxifen (E8284, Sigma-Aldrich, St. Louis, MO, USA) at day 1 for the gastric organoids and day 2 for the mammary organoids. The expression of tdTomato was then assessed to check for the proper induction of the organoids that were then processed for drugging at day 2 for the gastric organoids and day 4 for the mammary organoids. WT, *Cdh1^−/−^* and *Cdh1^−/−^/Tp53^−/−^* organoids were seeded as technical triplicates per drug concentration. After 48 h, pictures of the wells were taken, using Z-stack on a Cytation 5 imager (Biotek, Winooski, VT, USA). Pictures were then combined and focus-stacked, using a custom Python script. The area of the organoids was measured, using ImageJ, and normalized to the DMSO controls. The circularity of the *Cdh1^+/−^* organoids was measured by using ImageJ shape descriptor, with a value of 1 indicating a perfect circle (circularity = 4π(area/perimeter^2).

### 2.6. FACS

FACS was performed on human NCI-N87 cancer gastric cells and on mouse gastric organoids. NCI-N87 were seeded at 1.5 × 10^5^ or 2 × 10^5^ cells/well in a 6-well plate for the WT and *CDH1^−/−^* respectively. After 24 h, cells were then drugged for 48 h with 2.5 μM Entinostat, 0.31 μM Pracinostat, 0.63 μM Mocetinostat, 2.5 μM Vorinostat or their appropriate DMSO controls. To detect apoptosis, media and trypsinized cells were harvested and stained with propidium iodide (P4864, Sigma-Aldrich, St. Louis, MO, USA) and FITC-Annexin-V (#556420, BD Biosciences, San Jose, CA, USA) in Annexin Binding Buffer (0.01 M Hepes, 0.14 M NaCl, 0.25 mM CaCl_2_). Compensation controls were untreated/unstained, heat-treated (99 °C for 5 min), stained with propidium iodide, and Staurosporine (0.4 μM) stained with FITC-Annexin; and the positive control was Staurosporine stained with propidium iodide and FITC-Annexin. To detect proliferation, cells were harvested and fixed in 70% EtOH overnight at 4 °C. Cells were then washed with PFT buffer (1% FBS, 0.25% Triton in PBS) and incubated with 10 μg/mL Hoechst (H1399, Thermo Fisher Scientific, Waltham, MA, USA) and a rabbit Ki-67 antibody 1:200 (ab16667, Abcam, Cambridge, UK) diluted in PFT for 30 min. Cells were then washed with PFT and incubated for 30 min with goat anti-rabbit antibody 1:500 (A11008, Alexa Fluor 488, Invitrogen, Carlsbad, CA, USA). Compensation controls were untreated/unstained, Hoechst-stained and Ki-67-stained; and the positive control for proliferation inhibition was 0.5 μM Neratinib. For cell-cycle analysis, after fixation in 70% EtOH, cells were incubated 20 min in RTP buffer (0.05 mg/mL RNAse A, 0.1% Triton in PBS) at 37 °C. Cells were then labeled with propidium iodide (P4864, Sigma-Aldrich, St. Louis, MO, USA), and samples were then analyzed on a BD Fortessa Flow cytometer. FlowJo was used to analyze the different phase of the cell cycle, using Watson (pragmatic) model with unconstrained G1 and G2 peaks.

FACS was performed on WT and *Cdh1^+/−^* organoids after 48 h of drugging with entinostat, to assess for E-cadherin expression. Briefly, organoids were harvested and processed into single cells before fixation in 4% PFA for 15 min. Cells were then resuspended in 2% BSA–PBS containing the E-cadherin Alexa Fluor 647-conjugated antibody 1:200 (FAB7481R, R&D Systems, Minneapolis, MN, USA) for 30 min on ice. Cells were then washed in PBS and resuspended in 1% BSA–PBS. Samples were analyzed by using a BD Fortessa Flow cytometer.

### 2.7. Western Blot

WT, *Cdh1^−/−^* and *Cdh1^−/−^/Tp53^−/−^* organoids were seeded as single cells in a 24-well plate with 1000 cells per well. The organoids were induced at day 1 after seeding with endoxifen as for the drugging assay. At day 5, 4 wells per group were pooled together, and total cell protein was extracted in Triton-X lysis buffer (50 mM Tris, 150 mM NaCl, 1% Triton) containing 1× complete mini protease inhibitor tablet (11836170001, Sigma-Aldrich, St. Louis, MO, USA). Protein concentration was assessed by using the BCA protein assay kit (23225, Thermo Fisher Scientific, Waltham, MA, USA). Samples containing 20 μg of proteins were denatured in 4× Laemmli buffer (250 mM Tris, 8% SDS, 40% glycerol, 8% β-mercaptoethanol and 0.20% bromophenol blue), heated at 95 °C, separated on a 4–12% SDS–PAGE gel and transferred to a nitrocellulose membrane. The membranes were then blocked in 5% BSA/TBS-1%Tween for 1 h and incubated overnight at 4 °C in primary antibody goat E-Cadherin 1:1000 (AF748, R&D Systems, Minneapolis, MN, USA), rabbit Tp53 1:1000 (ab246550, Abcam, Cambridge, UK) or rabbit β-actin 1:2000 (A2066, Sigma-Aldrich, St. Louis, MO, USA). The following day, membranes were incubated for 1 h with their corresponding fluorescent secondary antibody: IRDye 800 CW Goat anti-Rabbit 1:10,000 (925-32211, LI-COR, Lincoln, NE, USA), IRDye 800 CW Donkey anti-Goat 1:10,000 (925-32214, LI-COR, Lincoln, NE, USA). Stripping was performed between Tp53 and Actin staining to avoid any double-staining. Fluorescent signal was assessed on an Odyssey Imaging System (LI-COR, Lincoln, NE, USA).

### 2.8. Immunofluorescence

For immunofluorescence, organoids were seeded as single cells on a Falcon 4-well Culture slide (FAL354114, In Vitro Technologies, Mt Wellington, Auckland, New Zealand) with 200 cells per well and were induced at day 1 with endoxifen as previously described. If required, organoids were drugged at day 2 for 48 h. At day 4 or 5, organoids were washed for 1 h with PBS and fixed in 1% PFA for 1 h, followed by blocking in 10% FHS/0.5% Triton in PBS. Organoids were then incubated for 3 h, at RT, with primary antibody E-Cadherin 1:100 (AF748, R&D Systems, Minneapolis, MN, USA), Tp53 1:100 (ab246550, Abcam, Cambridge, UK) or Ki-67 1:100 (ab1667, Abcam, Cambridge, UK), followed by a 2 h incubation with the according secondary antibody Donkey anti-Goat Alexa fluor 488 1:400 (A11055, Thermo Fisher Scientific, Waltham, MA, USA) or Goat anti-Rabbit Alexa Fluor 488 1:500 (A11008, Thermo Fisher Scientific, Waltham, MA, USA). Slides were then mounted with ProLong Gold Antifade Mounting with DAPI (P36935, Thermo Fisher Scientific, Waltham, MA, USA) and imaged with a Nikon A1+ inverted confocal laser scanning microscope and a Nikon DSiR2 color 16 MP camera (Otago Micro and Nanoscale Imaging, University of Otago). Organoids were imaged every 10 microns.

### 2.9. Statistical Analysis

All data are presented as the mean ± SEM. Analyses were performed by using Prism 8 software. Gaussian distribution was assessed by using the Shapiro–Wilk test, and the comparisons between two groups were performed by using multiple t-tests. Comparisons between WT, *Cdh1^−/−^* and *Cdh1^−/−^/Tp53^−/−^* organoids were performed by using 2-way ANOVA with Dunnett or Tukey multiple comparisons test. Correlation analysis between *CDH1* expression and HDACs were performed, using Spearman correlation followed by a Holm–Bonferroni test (adjusted *p*-value). Statistically significant results are labeled as * *p* < 0.05, ** *p* < 0.01 and *** *p* < 0.001.

## 3. Results

### 3.1. HDAC Classes Show Contrasting Correlations with CDH1 Expression

**Figure 1:**
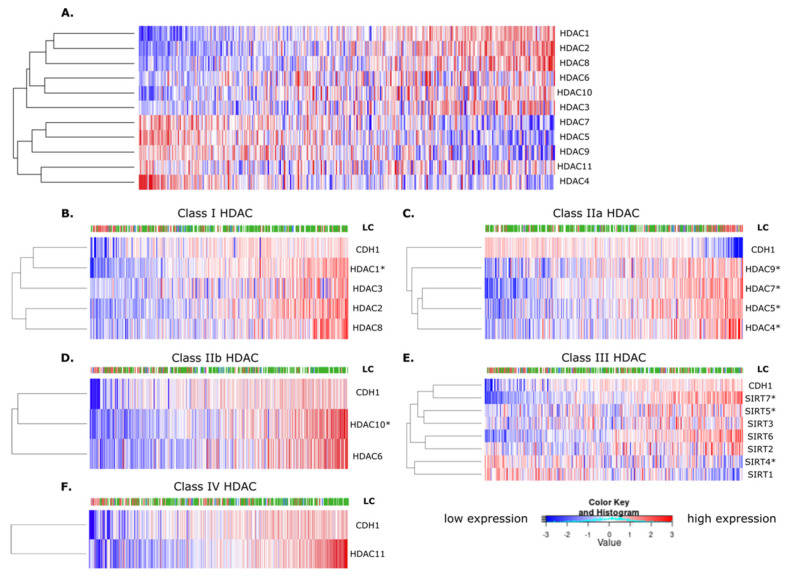
TCGA and STAD database highlight the correlation between HDACs and *CDH1* expression. The relationship between HDAC classes and *CDH1* expression from the Cancer Genome Atlas (TCGA) and the Stomach Adenocarcinoma (STAD) database was analyzed and the Spearman correlation was calculated between each HDAC and *CDH1* (detailed values in Table S4). A low expression is represented in blue, and a high expression in red. (**A**) Hierarchical clustering of zinc and NAD+-dependent HDACs. Correlation between HDACs and *CDH1* for (**B**) Class I HDAC (HDAC1, HDAC2, HDAC3 and HDAC8), (**C**) Class IIa HDAC (HDAC4, HDAC5, HDAC7 and HDAC9), (**D**) Class IIb (HDAC6 and HDAC10), (**E**) Class III HDAC (Sirtuins) and (**F**) Class IV HDAC (HDAC11). The HDACs with a significant correlation with *CDH1* expression are labeled with an asterisk (*). LC = Lauren Classification, representing the different gastric cancer subtypes: red, diffuse; green, intestinal; and blue, mixed subtypes.

To better understand the relationship between the different HDAC classes and *CDH1* expression in gastric cancer, we performed hierarchical clustering on gene-expression data from 415 gastric cancers (TCGA and STAD dataset). Notably, when comparing the zinc-dependent HDACs, the Class I and IIb HDACs showed an overall inverse relationship to the Class IIa and IV HDACs (Figure 1A). When compared directly to *CDH1* expression (Figure 1B–F and Spearman correlation Table S4), each of the Class IIa HDACs was significantly negatively correlated with *CDH1* expression, with a mean Spearman correlation of −0.23 (Figure 1C). Of the Class I and IIb HDACs, both HDAC1 and HDAC10 showed a significant positive correlation with *CDH1* (Figure 1B,D). In Class III, SIRT4 was negatively correlated with *CDH1* expression, whereas both SIRT5 and SIRT7 were positively correlated (Figure 1E). Finally, HDAC11 from Class IV was not correlated with *CDH1* expression (Figure 1F). Overall, these results illustrate the complex, but connected, relationships between the different HDAC classes and transcriptional patterns involving *CDH1* in gastric cancer. The correlations also suggest that targeting Class IIa HDACs might provide greater specificity for gastric cancers with low *CDH1* expression.

### 3.2. Breast and Gastric Cells Deficient for E-Cadherin Are Sensitive to HDAC Inhibitors

To confirm earlier observations of the enhanced inhibitory effect of HDAC inhibitors on E-cadherin-deficient cells [12], we treated an isogenic pair of MCF10A-wild-type (WT) and MCF10A-*CDH1^−/−^* cells with the pan-HDAC inhibitors entinostat, pracinostat, mocetinostat and vorinostat and confirmed the greater inhibition of MCF10A-*CDH1^−/−^* compared to the MCF10A-WT after 48 h of treatment (Figure S1 and Table S5). The observed inhibitory effects occurred at concentrations at which histone deacetylation inhibition has previously been observed in other cell lines [31,32]. A maximum decrease (with a *p*-value < 0.05) of 18% in the number of MCF10A-*CDH1^−/−^* cells compared to MCF10A-WT was observed for entinostat, 15% for pracinostat, 14% for mocetinostat and 13% for vorinostat. Other FDA-approved pan-HDAC inhibitors, namely belinostat, panobinostat and romidepsin, did not show any evidence of a synthetic lethal effect on the MCF10A isogenic cells (data not shown).

**Figure 2:**
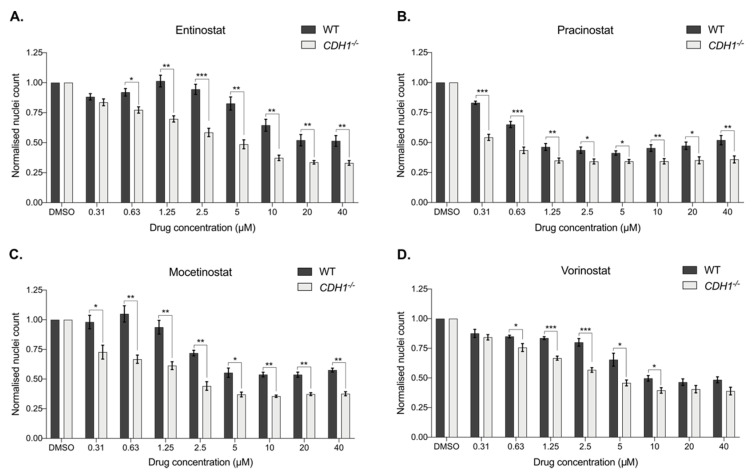
Pan-HDAC inhibitors have a synthetic lethal effect in gastric cancer cells lacking E-cadherin. A drugging experiment was performed on NCI-N87-WT and NCI-N87-*CDH1^−/−^* cells, and nuclei count was measured after 48 h. (**A**) Entinostat induced a synthetic lethal effect from 0.63 μM, with a maximum of 36% differences between both groups at 2.5 μM. (**B**) Effect of pracinostat was significant from 0.31 μM, with a maximum difference of 29%. (**C**) Mocetinostat was significant from 0.31 μM, with a maximum difference of 38%. (**D**) Vorinostat induced a synthetic lethal effect from 0.63 to 10 μM, with a maximum effect at 2.5 μM, with 23% differences between the two groups; *n* = 3–5 per compound. All the IC50 are summarized in Table S5. Statistically significant results are labeled as * *p* < 0.05, ** *p* < 0.01 and *** *p* < 0.001.

Next, drugging assays were performed on an isogenic cell line pair derived from gastric cancer cells in which *CDH1* deletion was generated by CRISPR-Cas9: NCI-N87-WT and NCI-N87-*CDH1^−/−^* [9] (Figure 2). Each of the four pan-HDAC inhibitors preferentially reduced the growth of *CDH1*-gastric-deficient cells compared to WT. NCI-N87 gastric cancer cells were less sensitive than the MCF10A breast cells to the pan-HDAC inhibitors tested, as demonstrated by a higher IC50 (Table S5), but the differential between the isogenic lines was more pronounced in NCI-N87 cells (NCI-N87-WT vs. NCI-N87- *CDH1^−/−^*: entinostat up to 36% (Figure 2A), pracinostat 29% (Figure 2B), mocetinostat 38% (Figure 2C) and vorinostat 23% (Figure 2D)).

### 3.3. Pan-HDAC Inhibitors Are More Robust to Genetic Background Than More Class-Specific HDAC Inhibitors

In order to identify any HDAC class-specific effects, we tested a panel of HDAC inhibitors with increased specificity (listed in Table S1). These HDAC inhibitors were first tested in the MCF10A isogenic pairs (Figure S2A). Of the 18 targeted HDAC inhibitors tested, only RGFP966 (HDAC3 inhibitor), LMK-235 (HDAC4 and 5 inhibitor), TH-34 (HDAC6, 8 and 10 inhibitor), SirReal2 (SIRT2 inhibitor) and LC-0296 (SIRT3 inhibitor) induced a synthetic lethal effect in this cell line pair with a significant decrease in the number of MCF10A-*CDH1^−/−^* cells compared to the MCF10A-WT cells after 72 h (Figure S2B). In this cell line, the inhibitory effect of a proportion of the targeted HDAC inhibitors was greater than the pan-HDAC inhibitors, with a maximum difference up to 25% for RGFP966, 39% for LMK-235, 27% for TH-34, 32% for SirReal2 and 35% for LC-0296.

In contrast to the isogenic MCF10A cells, the targeted HDAC inhibitors showed few synthetic lethal effects when tested on *CDH1* isogenic NCI-N87 cells. Romidepsin (HDAC1 and 2 inhibitor) was highly toxic to both NCI-N87 cell lines after 72 h (data not shown), but had a synthetic lethal effect up to 27.5% difference between the NCI-N87-*CDH1^−/−^* and NCI-N87-WT cells after only 24 h of treatment (Figure S3). Tasquinimod (HDAC4 inhibitor) presented a marginal synthetic lethal effect after 72 h (6%, Figure S3). Finally, Sis17 (HDAC11 inhibitor) also induced a difference in the number of NCI-N87-*CDH1^−/−^* compared to NCI-N87-WT cells (18.6% at 20 μM, Figure S3), but this observed difference was due to stimulation of NCI-N87-WT cell growth, not an increased toxicity for *CDH1^−/−^* cells. Two of the targeted HDAC inhibitors, TH34 (HDAC6, 8 and 10 inhibitor) and LC-0296 (SIRT3 inhibitor), induced a significant “reverse” synthetic lethal effect, with greater toxicity against the WT cells compared to the *CDH1^−/−^* cells after 72 h of treatment (Figure S3). Together, these results suggest that the simultaneous inhibition of multiple HDACs with pan-HDAC inhibitors is more likely to provide robust inhibition of E-cadherin-deficient cells across different genetic backgrounds than a more targeted approach.

### 3.4. Pan-HDAC Inhibitors Preferentially Induce Apoptosis and Decrease Proliferation in E-Cadherin-Deficient Cells

**Figure 3:**
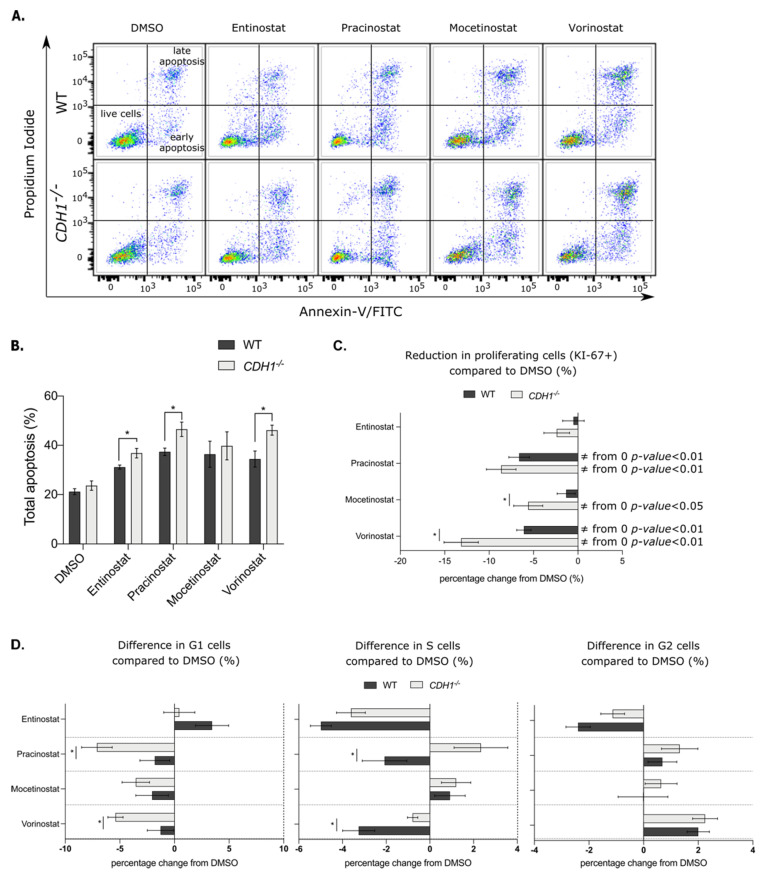
Pan-HDAC inhibitors preferentially induce apoptosis and decrease proliferation in gastric E-cadherin-deficient cells. Apoptosis, proliferation and cell cycle were analyzed in NCI-N87-*CDH1^−/−^* and NCI-N87-WT cells after 48 h of drugging with pan-HDAC inhibitors. (**A**) Apoptosis was analyzed by flow cytometry, using Annexin-V-FITC/propidium iodide staining. Annexin-V-FITC labels a membrane protein expressed by early apoptotic cells and propidium iodide stains nuclear DNA only accessible in apoptotic cells that have lost their membrane permeability (late apoptosis, *n* = 4–8). (**B**) Percentage of total apoptosis (early and late apoptosis combined) was increased in NCI-N87-*CDH1^−/−^* compared to NCI-N87-WT cells after 2.5 μM entinostat, 0.3 μM pracinostat and 2.5 μM vorinostat drugging (*n* = 3–5 per compound). (**C**) Total proliferation in NCI-N87-*CDH1^−/−^* and NCI-N87-WT cells was analyzed by flow cytometry after 48 h of drugging with pan-HDAC inhibitors. The difference in KI-67-positive cells for each drug was normalized compared to their respective DMSO. Mocetinostat (0.6 μM) and vorinostat (2.5 μM) preferentially and significantly decreased proliferation in NCI-N87-*CDH1^−/−^* cells compared to WT ones (*n* = 4–7); * = *p*-value < 0.05 for *CDH1^−/−^* vs. WT and ≠ from 0 for value statistically different to their respective DMSO (value of 0%). (**D**) Difference in percentage of cells in each phase of the cell cycle compared to their respective DMSO by flow cytometry, using propidium iodide staining (*n* = 4–5). Pracinostat and vorinostat significantly decreased the percentage of NCI-N87-*CDH1^−/−^* cells in G1 phase and rationally increased the proportion of cells in the S phase.

To assess if the increased sensitivity of *CDH1^−/−^* cells to the pan-HDAC inhibitors was due to a cytotoxic or cytostatic mechanism, we first measured the percentage of total apoptosis by Annexin-V-FITC/propidium iodide flow cytometry. Cells were drugged for 48 h, using the drug concentrations that had the greatest synthetic lethal effect in the NCI-N87 nuclei count assay (Figure 3A). We observed a significant increase in apoptosis in NCI-N87-*CDH1*^−/−^ cells compared to NCI-N87-WT cells for three of the pan-HDAC inhibitors tested, with relative increases of 18% for 2.5 μM entinostat, 25% for 0.3 μM pracinostat and 34% for 2.5 μM vorinostat (Figure 3B).

Next, proliferation was assessed by flow cytometry, using KI-67 labeling (Figure 3C). The percentage of proliferating cells (KI-67 positive) was measured after 48 h of drugging and normalized to their respective control (DMSO treated cells). DMSO alone did not cause any significant difference in the total amount of cells positive for KI-67 in the two cell lines (average of 90% proliferative cells for NCI-N87-WT and 84% for NCI-N87-*CDH1^−/−^* cells, *p*-value = 0.06, data not shown). Although entinostat (2.5 μM) did not significantly change proliferation of WT or *CDH1^−/−^* NCI-N87 cells, pracinostat (0.3 μM) and vorinostat (2.5 μM) significantly decreased proliferation of both cell lines (*p*-value < 0.01, Figure 3C). Mocetinostat (0.6 μM) did not impact proliferation of WT cells, but significantly reduced the proliferation of NCI-N87-*CDH1^−/−^* cells (*p*-value < 0.05, Figure 3C). NCI-N87-*CDH1^−/−^* cells were more sensitive to the anti-proliferative effects of mocetinostat and vorinostat compared to NCI-N87-WT cells with a relative decrease of proliferation of 11% and 15%, respectively (Figure 3C).

The potential cytostatic effect of pan-HDAC inhibitors on the NCI-N87 isogenic cells was also examined by cell-cycle analysis. After 48 h of drugging, pracinostat and vorinostat significantly reduced the percentage of NCI-N87-*CDH1^−/−^* cells in G1 phase compared to NCI-N87-WT cells and increased the proportion of cells in the S phase (Figure 3D). Entinostat and mocetinostat did not differentially impact the cell cycle in WT and NCI-N87 cells deficient for E-cadherin. Together, the results indicate that the increased sensitivity of *CDH1^−/−^* cells to pan-HDAC inhibitors is due to both cytostatic and cytotoxic effects, but these effects are not consistent between the four drugs tested.

### 3.5. The Characterization of E-Cadherin-Deficient Mouse Gastric Organoids

To test the pan-HDAC inhibitors in a more physiologically relevant model, we generated mouse gastric organoids originating from transgenic, endoxifen-inducible Cre-lox mice under the control of the CD44 stem cell promoter. Each organoid construct included homozygous floxed alleles encoding the red fluorescent marker protein tdTomato. WT organoids were generated from the stomachs of *CD44*-Cre/tdTomato^(fl/fl)^ mice. *Cdh1^−/−^* organoids were generated from *CD44*-Cre/*Cdh1*^(fl/fl)^/tdTomato^(fl/fl)^ mice with loxP sites flanking exons 6 to 10 of *Cdh1*; double *Cdh1/Tp53* knockout organoids were generated from CD44-Cre/*Cdh1*^(fl/fl)^/*Tp53*^(fl/fl)^/tdTomato^(fl/fl)^ mice with loxP sites flanking exons 6 to 10 of *Cdh1* and exons 2 to 10 of *Tp53*. *CDH1* and *TP53* are the most frequently mutated genes in human DGC, occurring in 33% and 34% of tumors, respectively [33], and knockout of both these genes under the control of the *Atp4b* promoter has previously been shown to lead to metastatic gastric cancer [34].

**Figure 4:**
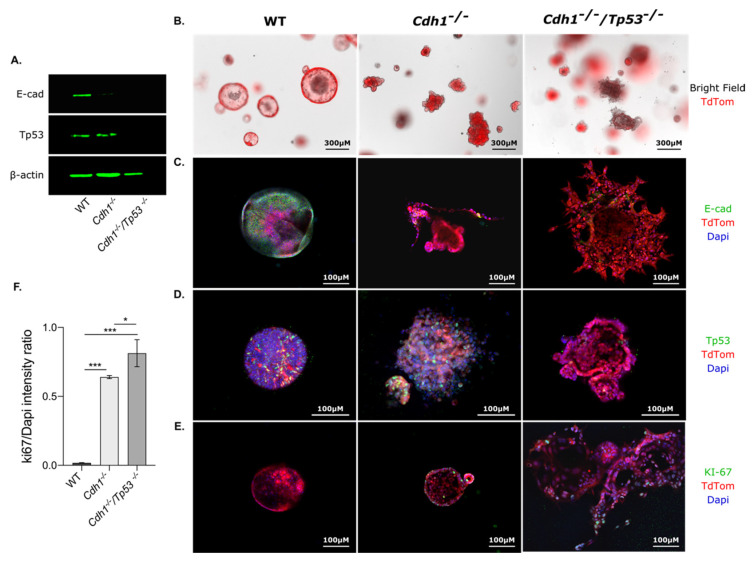
*Cdh1* and *Tp53* inactivation disrupt gastric organoids morphology. Organoids were generated using inducible Cre-lox mice. WT organoids express an inducible tdTomato fluorescence following Cre induction. *Cdh1^−/−^* organoids exhibit tdTomato expression and *Cdh1* deletion while *Cdh1^−/−^*/*Tp53^−/−^* organoids exhibit tdTomato expression associated with *Cdh1* and *Tp53* deletion when induced with endoxifen. Organoids were induced at day 1 post-seeding followed by Western blot and immunofluorescences at day 6. (**A**) Western blot was performed on protein extracted from WT, *Cdh1^−/−^* and *Cdh1^−/−^*/*Tp53^−/−^* organoids. E-cadherin staining was observed at 110 kDa, Tp53 at 53 kDa and β-actin at 45 kDa. (**B**) Bright field and tdTomato expression were assessed after 5 days of induction with endoxifen. WT organoids maintained a circular morphology while both *Cdh1^−/−^* and *Cdh1^−/−^*/*Tp53^−/−^* organoids displayed a loss of circularity with the presence of evaginations. (**C**) Immunofluorescence on WT, *Cdh1^−/−^* and *Cdh1^−/−^*/*Tp53^−/−^* organoids with E-cadherin stained in green, tdTomato in red and DAPI in blue for nuclear staining. The absence of E-cadherin was confirmed in both *Cdh1^−/−^* and *Cdh1^−/−^*/*Tp53^−/−^* organoids. (**D**) Immunofluorescence with Tp53 staining in green. Tp53 was present in the WT and *Cdh1^−/−^* organoids and absent in the *Cdh1^−/−^*/*Tp53^−/−^* organoids. (**E**) KI-67 to label for proliferation was performed at day 6 post-seeding. KI-67 (in green) was absent in the WT organoids at day 6 indicating an arrest of proliferation whereas *Cdh1^−/−^* and *Cdh1^−/−^*/*Tp53^−/−^* organoids still presented cells in proliferation. (**F**) The intensity ratio between KI-67 and DAPI staining was measured every 10 microns on whole WT, *Cdh1^−/−^* and *Cdh1^−/−^*/*Tp53^−/−^* organoids by confocal microscopy. Statistically significant results are labeled as * *p* < 0.05 and *** *p* < 0.001.

We first confirmed the deletion of *Cdh1* and/or *Tp53* in our gastric organoid models using Western blotting of proteins extracted on day 6 post-seeding, five days after induction with endoxifen (Figure 4A and Figure S4). Immunofluorescence was then performed on whole organoids 5 days after induction. TdTomato staining was observed in WT, *Cdh1^−/−^* and *Cdh1^−/−^*/*Tp53^−/−^* organoids, confirming the induction of Cre in our model (Figure 4B). While the WT organoids maintained a simple circular morphology 5 days after induction, both *Cdh1^−/−^* and *Cdh1^−/−^*/*Tp53^−/−^* organoids showed a disrupted irregular shape, with frequent evaginations and cells displaced from the organoid surface (Figure 4B–E). This disruption of morphology was particularly pronounced in the *Cdh1^−/−^*/*Tp53^−/−^* organoids.

Immunofluorescence on WT, *Cdh1^−/−^* and *Cdh1^−/−^*/*Tp53^−/−^* organoids with E-cadherin antibodies showed that, as expected, E-cadherin was strongly expressed in WT organoids and largely absent from the *Cdh1^−/−^* and *Cdh1^−/−^*/*Tp53^−/−^* organoids (Figure 4C). Immunofluorescence also detected Tp53 expression in a population of cells in the WT and *Cdh1^−/−^* organoids and confirmed its near complete loss from the *Cdh1^−/−^*/*Tp53^−/−^* organoids (Figure 4D). Proliferation was also assessed at day 4 by KI-67 antibody staining and showed proliferation in WT, *Cdh1^−/−^* and *Cdh1^−/−^*/*Tp53^−/−^* organoids (data not shown). However, at day 6, only *Cdh1^−/−^* and *Cdh1^−/−^*/*Tp53^−/−^* organoids contained proliferating cells (Figure 4E). Moreover, the intensity ratio between KI-67 and DAPI nuclear staining was measured every 10 microns on the whole organoids and then averaged (Figure 4F). The ratio was significantly higher in *Cdh1^−/−^* and *Cdh1^−/−^*/*Tp53^−/−^* organoids compared to WT. These results are consistent with previous studies that have shown that inactivation of E-cadherin and Tp53 increases the proliferation rate in both gastric and mammary epithelial cancer cells [35,36,37].

### 3.6. Pan-HDAC Inhibitors Preferentially Target Organoids Lacking E-Cadherin Expression

#### 3.6.1. Gastric Organoids

**Figure 5:**
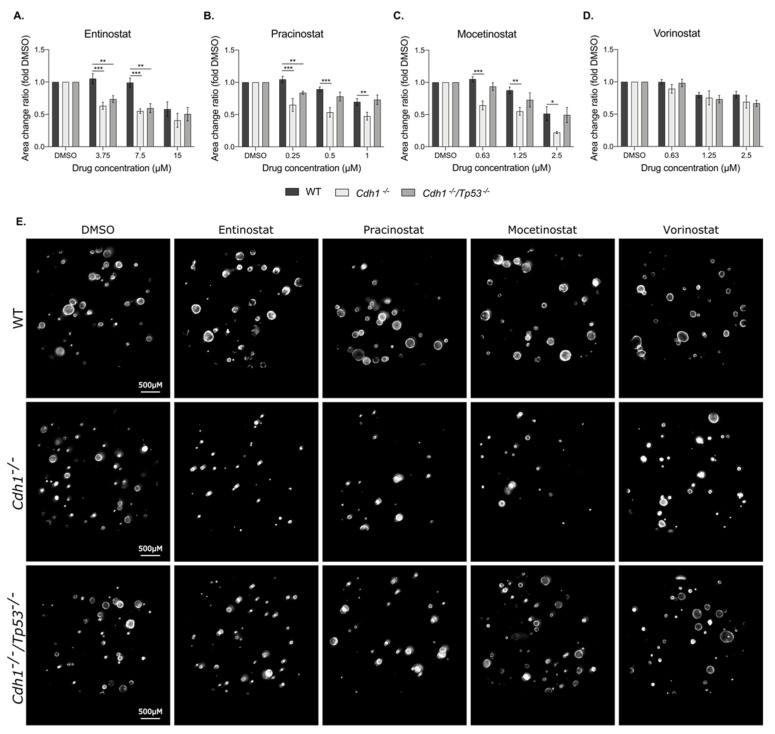
E-cadherin-deficient gastric organoids are more sensitive to pan-HDAC inhibitors. WT, *Cdh1^−/−^* and *Cdh1^−/−^*/*Tp53^−/−^* organoids were drugged for 48 h with the different pan-HDAC inhibitors, and the area of the organoids was then measured and normalized to their respective vehicle controls. RFP fluorescence was used to detect tdTomato and determine the area of the organoids, using Cytation 5 imager (Biotek, Winooski, VT, USA). (**A**) Entinostat induced a synthetic lethal effect with a maximum of 44% differences between WT organoids and *Cdh1^−/−^* ones and 40% for the *Cdh1^−/−^*/*Tp53^−/−^* organoids. (**B**) Pracinostat induced an SL effect up to 40% and 20% for the *Cdh1^−/−^* and the *Cdh1^−/−^*/*Tp53^−/−^* organoids respectively. (**C**) Mocetinostat preferentially targeted E-cadherin-deficient cells with a maximum of 39% decrease in *Cdh1^−/−^* area compared to WT ones and had no effect for *Cdh1^−/−^*/*Tp53^−/−^* organoids. (**D**) Vorinostat did not present any significant synthetic lethal effect between the WT and the E-cadherin-deficient gastric organoids. *n* = 3–5 per compound. All the IC50 are summarized in Table S5. Statistically significant results are labeled as * *p* < 0.05, ** *p* < 0.01 and *** *p* < 0.001. (**E**) Picture representation of WT, *Cdh1^−/−^* and *Cdh1^−/−^*/*Trp53^−/−^* organoids after 7.5 μM entinostat, 0.25 μM pracinostat, 0.63 μM mocetinostat and 0.63 μM vorinostat.

Gastric organoids were drugged with pan-HDAC inhibitors for 48 h before their area was measured, and the averages were normalized to their respective DMSO-treated controls (Figure 5). As previously observed in MCF10A and NCI-N87 cells, entinostat preferentially inhibited the growth of cells lacking E-cadherin, with a decrease in area of up to 44% at 7.5 µM after 48 h of treatment in *Cdh1^−/−^* organoids and 40% in *Cdh1^−/−^*/*Tp53^−/−^* organoids compared to WT (Figure 5A,E and Table S5). Pracinostat also induced a synthetic lethal effect with a maximum difference at 0.5 µM of 40% in the area of *Cdh1^−/−^* compared to WT organoids (Figure 5B,E). For the *Cdh1^−/−^*/*Tp53^−/−^* organoids, a significant synthetic lethal effect was only observed at 0.25 µM, with a difference of 20% compared to the WT organoids. Mocetinostat decreased growth of *Cdh1^−/−^* organoids by 39% at 0.63 µM relative to the WT organoids, but no statistically significant synthetic lethal effect was observed for the *Cdh1^−/−^*/*Tp53^−/−^* organoids (Figure 5C,E). Vorinostat showed no preferential growth inhibition of E-cadherin-negative organoids, regardless of the *Tp53* genotype (Figure 5D,E). Together, these results suggest that *Tp53* mutation is a marker of partial resistance to pracinostat and mocetinostat, but not entinostat, which retains a strong synthetic lethal effect in gastric organoids with both *Cdh1^−/−^ and Cdh1^−/−^/Tp53^−/−^* genetic backgrounds.

#### 3.6.2. Mammary Organoids

**Figure 6:**
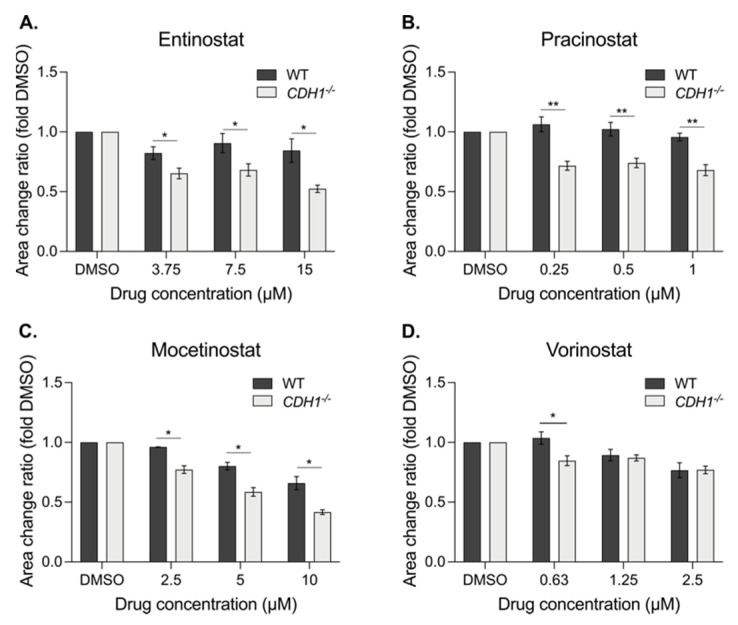
E-cadherin-deficient mammary organoids are more sensitive to pan-HDAC inhibitors. WT and *Cdh1^−/−^* mammary organoids were generated from the same Cre-lox mice than the gastric organoids and drugged with the pan-HDAC inhibitors. The mammary organoids area were then assessed after 48 h of drugging and normalized to their respective DMSO. (**A**) Entinostat induced a synthetic lethal effect, with a maximum of 32% differences at 15 µM, between WT and *Cdh1^−/−^* organoids. (**B**) Pracinostat decreased *Cdh1^−/−^* growth up to 35% at 0.25 µM compared to the WT. (**C**) Mocetinostat induced an SL effect, with a maximum of 24% decrease at 10 µM, in the *Cdh1^−/−^* area compared to WT mammary organoids. (**D**) Vorinostat presented an SL effect at the lowest concentration, with a difference of 19% between the *Cdh1^−/−^* and WT organoids; *n* = 3–5 per compound. All the IC50 are summarized in Table S5. Statistically significant results are labeled as * *p* < 0.05 and ** *p* < 0.01.

To further confirm the efficacy of the pan-HDAC inhibitors on E-cadherin-deficient cells, mammary organoids from the same *CD44-Cre/Cdh1^(fl)/(fl)^*/tdTomato mice were generated and drugged by following a similar protocol to the gastric organoids [30]. As previously observed in the gastric organoids, entinostat, pracinostat and mocetinostat preferentially reduced the growth of E-cadherin-deficient mammary organoids compared to the WT, with a decrease in area up to 32% for 15 µM entinostat, 35% for 0.25 µM pracinostat and 24% for 10 µM mocetinostat (Figure 6A–C and Table S5). Similar to the gastric organoids, vorinostat showed little evidence of a *Cdh1* synthetic lethal effect on the mammary organoids (Figure 6D).

### 3.7. Entinostat Promotes E-Cadherin Expression in Organoids Heterozygous for Cdh1 Mutation

The growth-inhibition experiments described above focused on achieving chemoprevention in HDGC by inhibiting the survival of the *CDH1*-null multifocal stage T1a SRCCs that are present in *CDH1* germline mutation carriers. An alternative to this approach is to exploit the high frequency with which the second *CDH1* allele is inactivated epigenetically in these foci and use drugs to re-express it [2,38]. Re-expression of the epigenetically silenced *CDH1* allele would be predicted to restore adherens junctions and associated signaling, preventing the generation of *CDH1*-null gastric epithelial cells, which can be displaced into the *lamina propria,* beyond the influence of normal regulatory cues. Pan-HDAC inhibitors, including entinostat, pracinostat, mocetinostat and vorinostat, have previously been shown to activate *CDH1* expression [39,40], raising the possibility that these drugs could both eliminate established stage T1a SRCCs and prevent their formation.

**Figure 7:**
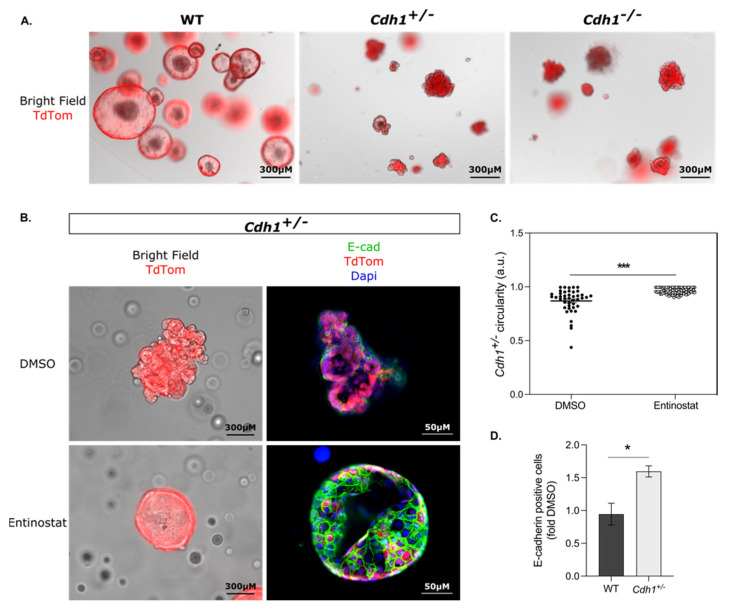
Entinostat increases E-cadherin expression in *Cdh1^+/−^* organoids, leading to rounded morphology. *Cdh1^+/−^* organoids were induced at day 1 followed by drugging at day2 with entinostat or DMSO for 48 h. (**A**) Bright field and tdTomato expression were assessed at day 4 in WT, *Cdh1^+/−^* and *Cdh1^−/−^* organoids. *Cdh1* decrease of expression was associated with a loss of circularity and the presence of evagination. (**B**) Immunofluorescence was performed on *Cdh1^+/−^* organoids following 48 h of treatment with entinostat or its respective DMSO concentration. Bright field and tdTomato showed a maintenance of a circular morphology following entinostat treatment. Immunofluorescence on *Cdh1^+/−^* organoids was performed with E-cadherin stained in green, tdTomato in red and DAPI in blue for nuclear staining. A decrease of E-cadherin was observed after induction in the *Cdh1^+/−^* DMSO-treated organoids, whereas entinostat treated *Cdh1^+/−^* organoids maintained a strong E-cadherin expression. (**C**) Circularity of the *Cdh1^+/−^* organoids was measured after DMSO or entinostat drugging, with a value of 1 representing a perfect circle (*n* = 43–97 organoids analyzed). (**D**) E-cadherin expression after entinostat drugging was measured by flow cytometry for WT and *Cdh1^+/−^* gastric organoids and normalized to their respective DMSO (*n* = 4). Entinostat treatment increased the expression of E-cadherin in the *Cdh1^+/−^* organoids. Statistically significant results are labeled as * *p* < 0.05 and *** *p* < 0.001.

We had previously observed that *Cdh1^+/−^* heterozygote gastric organoids generated from *CD44*-Cre/*Cdh1^(^*^fl/WT)^/tdTomato mice showed similar morphology to gastric organoids developed from *CD44*-Cre/*Cdh1^(^*^fl/fl)^/tdTomato mice in which both *Cdh1* alleles had been inactivated (Figure 7A). Immunofluorescence with anti-E-cadherin antibodies confirmed that a high proportion of cells in the *Cdh1* heterozygous organoids were E-cadherin-null (Figure 7B), presumably due to the epigenetic silencing of the wild-type *Cdh1* allele during the development of the organoids, similar to the natural history of HDGCs stage T1a gastric SRCCs [2]. To test the effect of entinostat on these *Cdh1* heterozygote organoids, *Cdh1^+/−^* organoids were treated with entinostat or DMSO control 24 h after endoxifen induction. Forty-eight hours later, strong tdTomato expression was observed in these *Cdh1^+/−^* organoids, demonstrating the activation of the Cre recombinase (Figure 7B). The DMSO-treated organoids displayed a disrupted shape and the presence of evaginations typical of *Cdh1*-null organoids. In contrast, entinostat treatment prevented the formation of evaginations and the organoids maintained the circular shape characteristic of WT organoids. Our analysis of the morphology of these organoids confirmed an increase circularity (characterized by a value of 1) with entinostat compared to DMSO (Figure 7C). Immunofluorescence demonstrated that E-cadherin was strongly expressed in the entinostat-treated *Cdh1*^+/−^ organoids compared to the same organoids treated with DMSO (Figure 7B). To confirm this effect, the expression of E-cadherin was verified by flow cytometry in WT and *Cdh1^+/−^* organoids after drugging (Figure 7D). The E-cadherin level was unchanged in the WT organoids following entinostat treatment, as compared to DMSO (fold change = 0.94, *p*-value = 0.8). However, E-cadherin expression was significantly increased with entinostat in the *Cdh1^+/−^* organoids (fold change of 1.60, *p*-value < 0.001). Together, these results demonstrate that entinostat is increasing the expression of the WT allele in heterozygous *Cdh1*^+/−^ organoids, leading to normalized organoid morphology.

## 4. Discussion

By virtue of their central role in transcriptional regulation, it is unsurprising that the expression of HDACs is dysregulated in cancer, including breast and gastric tumors [41]. However, we were interested in understanding if any HDACs were co-regulated with *CDH1* in gastric cancer. We first examined the correlation between individual HDACs and *CDH1* expression in gastric tumors. Of the 18 HDACs, half of them were significantly correlated with *CDH1* expression, including four HDACs that were positively correlated (HDAC1, HDAC10, SIRT5 and SIRT7) and five that were negatively correlated (HDAC4, HDAC5, HDAC7, HDAC9 and SIRT4). These correlations may reflect the key role E-cadherin plays in major transcriptional patterns, particularly in the epithelial–mesenchymal transition [35].

HDAC inhibitors exert their anticancer effects through a range of cytotoxic or cytostatic mechanisms that are likely to be context dependent, including cell-cycle arrest, apoptosis and promotion of a terminally differentiated state [17,42]. We observed strong pro-apoptotic effects for each of entinostat, pracinostat, mocetinostat and vorinostat in the NCI-N87 cell lines. Notably, the percentage of cells that were apoptotic was higher in the NCI-N87-*CDH1^−/−^* line compared to WT cells, following treatment with each drug, suggesting that the increased sensitivity of *CDH1*-deficient cells to HDAC inhibitors is mediated, at least in part, by increased cytoxicity of the HDAC inhibitors in these cells. The four HDAC inhibitors also had a cytostatic effect on the isogenic NCI-N87 cells, but it was less pronounced than the pro-apoptotic effect. However, as observed with the apoptotic markers, the inhibitory effect was amplified in the *CDH1*-deficient cells, even though this increase was only significant for vorinostat and mocetinostat. Although other mechanisms such as autophagic apoptosis [25] or ferroptosis [25,43] may also be involved, these results show that the pan-HDAC inhibitors are acting preferentially on *CDH1*-deficient cells through both cytotoxic and cytostatic mechanisms, as also observed in other cell lines [27,44,45].

To validate the effects of the pan-HDAC inhibitors in a more complex gastric model, entinostat, pracinostat, mocetinostat and vorinostat were tested on inducible mouse-derived gastric organoids with either WT, *Cdh1^−/−^* or *Cdh1^−/−^/Tp53^−/−^* genetic backgrounds. Organoid models better reproduce the epithelial cell’s local environment and drug responses correlate well with in vivo effects [46]. Immunofluorescence on these organoids showed that the loss of *Cdh1* was associated with a disrupted morphology, evaginations and higher proliferation than control organoids. The addition of a *Tp53* mutation aggravated the irregular shape and the number of cells displaced from the organoid following loss of *Cdh1* expression. These results were consistent with previous studies that showed that E-cadherin and Tp53 inactivation are associated with an increase in proliferation, migration and invasiveness of cells [35,47].

Of the four pan-HDAC inhibitors tested, each of entinostat, pracinostat and mocetinostat exhibited significant synthetic lethal effects in the E-cadherin-deficient gastric and mammary gland organoids compared to WT. However, the additional deletion of *Tp53,* the most commonly mutated gene in DGC, in the gastric organoids strongly reduced this effect with pracinostat and mocetinostat, but not entinostat. Notably, entinostat has generally been well tolerated in clinical trials, with most side effects being mild to moderate and manageable with supportive care [48]. The differential inhibition between wild-type and *CDH1*-null cells was frequently statistically significant at sub-micromolar concentrations and consistent with the effective concentrations observed in numerous cancer cell lines [17,48,49].

Before testing entinostat in a chemoprevention clinical trial, it will be necessary to first demonstrate efficacy in an animal model. We recently developed a novel mouse model with tamoxifen-inducible inactivation of *Cdh1* and *Tp53* that develops diffuse type gastric cancers in ~3 months (unpublished data). Candidate chemoprevention drugs could be tested in this model in mice with established cancers, but also in others which would be treated with drug 2–4 weeks after tamoxifen-induction, with the tumor burden being quantified a further 8–10 weeks later.

Chemoprevention of HDGC can be achieved by either targeting the early stage T1a SRCC foci before they have acquired the capacity for invasion beyond the *muscularis mucosae*, or by reducing the risk of epigenetic silencing of the second *CDH1* allele, leading to complete loss of E-cadherin expression and migration of epithelial cells into the *lamina propria*. Our data suggest that entinostat, in contrast with other potential chemoprevention drugs [9,10], may be able to reduce cancer risk by both promoting apoptosis in established *CDH1*-null foci and preventing the initiation of new foci by maintaining the integrity of the epithelial plane.

We predict that the sub-millimeter median size of the HDGC stage T1a SRCC foci [50] and their genetic homogeneity (i.e., only *CDH1* mutations) will allow the use of chemoprevention drug doses that are well tolerated. Moreover, we predict that the relative indolence of these early cancers will enable treatment schedules that minimize the risk of accumulated toxicities and, at the same time, make compliance more likely (e.g., repeated treatment every 1–2 years). The small size and indolence of the HDGC stage T1a foci sets these cancers apart from advanced highly mutated cancers, which require large pharmacological windows and aggressive treatment strategies. Reductions in drug dose may also be enabled by the identification of synergistic combinations between entinostat and other synthetic lethal drugs. Methods to deliver drugs directly to the gastric mucosa would also be anticipated to minimize many of the side effects associated with systemic treatment. Ideally, drugs could be delivered to the mucosa at the same time as annual surveillance endoscopy. Our data showing drug efficacy in breast MCF10A isogenic cells and mouse mammary organoids also suggest that *CDH1* synthetic lethal drugs, such as entinostat, may also be effective in reducing the risk of LBC and lobular carcinoma in situ (LCIS) in HDGC families. Moreover, given the high frequency of somatic *CDH1* mutations in sporadic DGC, LCIS and LBC and their association with poor prognosis [51,52], these drugs may also be useful for the treatment of sporadic cancers.

The molecular vulnerability that sensitizes *CDH1*-null cells to pan-HDAC inhibitors is unclear. Although the effect may be related to a specific histone or non-histone target, the maintenance of a synthetic lethal effect with multiple HDAC inhibitors, both specific and pan-inhibitors, suggests that their effect may be more general, reflecting a shift in the balance of inputs into major cell survival pathways in *CDH1*-null cells, such as the PI3K/AKT, RAS and MAPK signaling pathways.

In conclusion, our study has demonstrated that E-cadherin-deficient gastric and breast cells are more sensitive to HDAC inhibitors than their wild-type isogenic pairs and suggests that that pan-HDAC inhibitors—in particular, entinostat—may have a role in the chemoprevention of HDGC and the treatment of sporadic LBC and DGC.

## 5. Conclusions

We have determined that pan-HDAC inhibitors preferentially target cells lacking E-cadherin expression in breast cells, gastric cancer cells and mouse-derived gastric and mammary organoids. Of all the pan-HDAC inhibitors tested, entinostat showed the most promising results, with a synthetic lethal effect in all our models. These results showed that pan-HDAC inhibitors could lead to new a chemoprevention and/or treatment strategy for HDGC patients with *CDH1* mutations.

## Data Availability

The data presented in this study are available on request from the corresponding author.

## Supplementary Materials

The following supporting information can be downloaded online at https://www.mdpi.com/article/10.3390/cancers14010175/s1. Figure S1: MCF10A E-cadherin-deficient cells are more sensitive to pan-HDAC inhibitors than normal MCF10A. Figure S2: Specific HDAC inhibitors induce a synthetic lethal effect in MCF10A deficient for E-cadherin. Figure S3: Specific HDAC inhibitors have less effect on NCI-N87 cells compared to pan-HDAC inhibitors. Figure S4: E-cadherin and Tp53 expression in WT and E-cadherin-deficient organoids. Table S1: List of the different compounds used for the drugging experiment, along with their respective targets. Table S2: Gastric organoid complete media. Table S3: Mammary organoid complete media. Table S4: Adjusted *p*-value of the Spearman correlation between HDACs and *CDH1* expression. Table S5: IC50 of the different pan-HDAC inhibitors obtained in our 2D and 3D models.
