# Supplementary material for: E-Cadherin-Deficient Epithelial Cells Are Sensitive to HDAC Inhibitors
Source: Cancers (Basel). 2021 Dec 30;14(1):175. doi: 10.3390/cancers14010175 (PMC8749989; doi:10.3390/cancers14010175)
Supplement: Supplementary file 1 [file cancers-14-00175-s001.zip › cancers-1517557-supplementary.pdf]

cancers

MDPI

Article

E-cadherin-deficient epithelial cells are sensitive to HDAC inhibitors.

Supplementary data

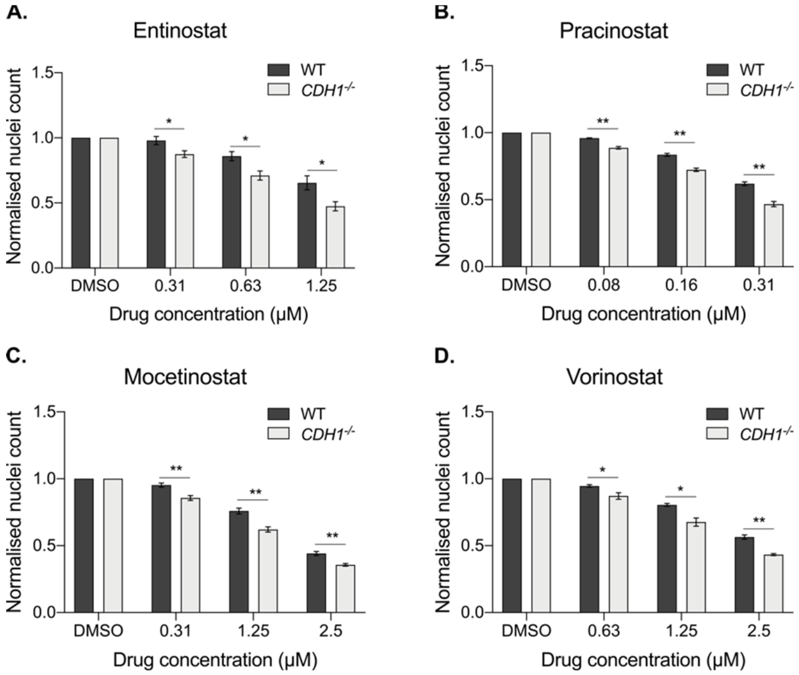

Figure S1. MCF10A E-cadherin-deficient cells are more sensitive to pan-HDAC inhibitors than WT MCF10A. MCF10A-WT and MCF10A-*CDH1*$^{-/-}$ cells were drugged with pan-HDAC inhibitors and cell number was assessed after 48h. The IC50 was measured for each pan-HDAC inhibitors and reported in table S2. **A.** Entinostat, **B.** Pracinostat, **C.** Mocetinostat and **D.** Vorinostat have a synthetic lethal effect with a greater inhibition of MCF10A-*CDH1*$^{-/-}$ cells compared to the WT. n=3-4 per compound. Statistically significant results are labeled as * $p < 0.05$ and ** $p < 0.01$.

A.

Compound	Targets	IC50
Romidepsin	HDAC1<br>HDAC2	IC50_WT, *CDH1*$^{-/-}$ > 93.38nM
Santacruzamate A	HDAC2	IC50_WT, *CDH1*$^{-/-}$ > 1µM
RGFP966	HDAC3	IC50_WT, *CDH1*$^{-/-}$ > 30µM *
Tasquinimod	HDAC4	IC50_WT, *CDH1*$^{-/-}$ > 40µM
LMK-235	HDAC4<br>HDAC5	IC50_WT > 20µM, *CDH1*$^{-/-}$ = 0.4 µM<br>***
TMP269	HDAC4<br>HDAC5<br>HDAC7<br>HDAC9	IC50_WT, *CDH1*$^{-/-}$ > 10µM
Tubastatin A	HDAC6	IC50_WT, *CDH1*$^{-/-}$ > 5µM
PCI-34051	HDAC8	IC50_WT, *CDH1*$^{-/-}$ > 5µM
TH34	HDAC6<br>HDAC8<br>HDAC10	IC50_WT > 20µM, *CDH1*$^{-/-}$ = 16µM<br>***
Sis17	HDAC11	IC50_WT = 25µM, *CDH1*$^{-/-}$ = 28µM
Ex527	SIRT1	IC50_WT, *CDH1*$^{-/-}$ > 40µM
SirReal2	SIRT2	IC50_WT = 35µM, *CDH1*$^{-/-}$ = 29µM *
LC-0296	SIRT3	IC50_WT > 80µM, *CDH1*$^{-/-}$ = 65µM *
Sirt6in5	SIRT6	IC50_WT, *CDH1*$^{-/-}$ > 100µM

B.

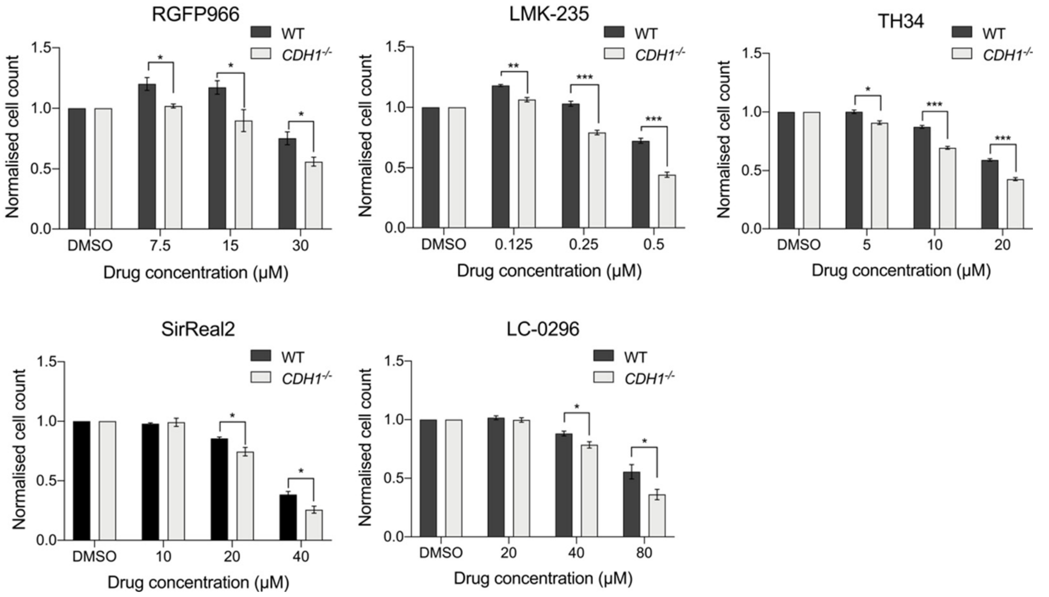

Figure S2. Specific HDAC inhibitors induce a synthetic lethal effect in MCF10A deficient for E-cadherin. MCF10A-WT and MCF10A-*CDH1*$^{-/-}$ cells were treated with specific HDAC inhibitors for 24h (Romidepsin) or 72h (for the other compounds). **A.** On the table, each compound is associated with its corresponding targets. A total of 8 concentrations has been tested for each compound and the IC50 have been measured if possible and reported in the table. The concentration of the compounds used in this experiment were determined following the literature recommendation to specifically inhibit certain HDAC. The *p-value* was calculated and reported on the table, *p-value* < 0.05 = *, *p-value* < 0.001 = ***. **B.** Graphs representing the drugging results for the compounds with a significant synthetic lethal effect. n=3-5 per compound.

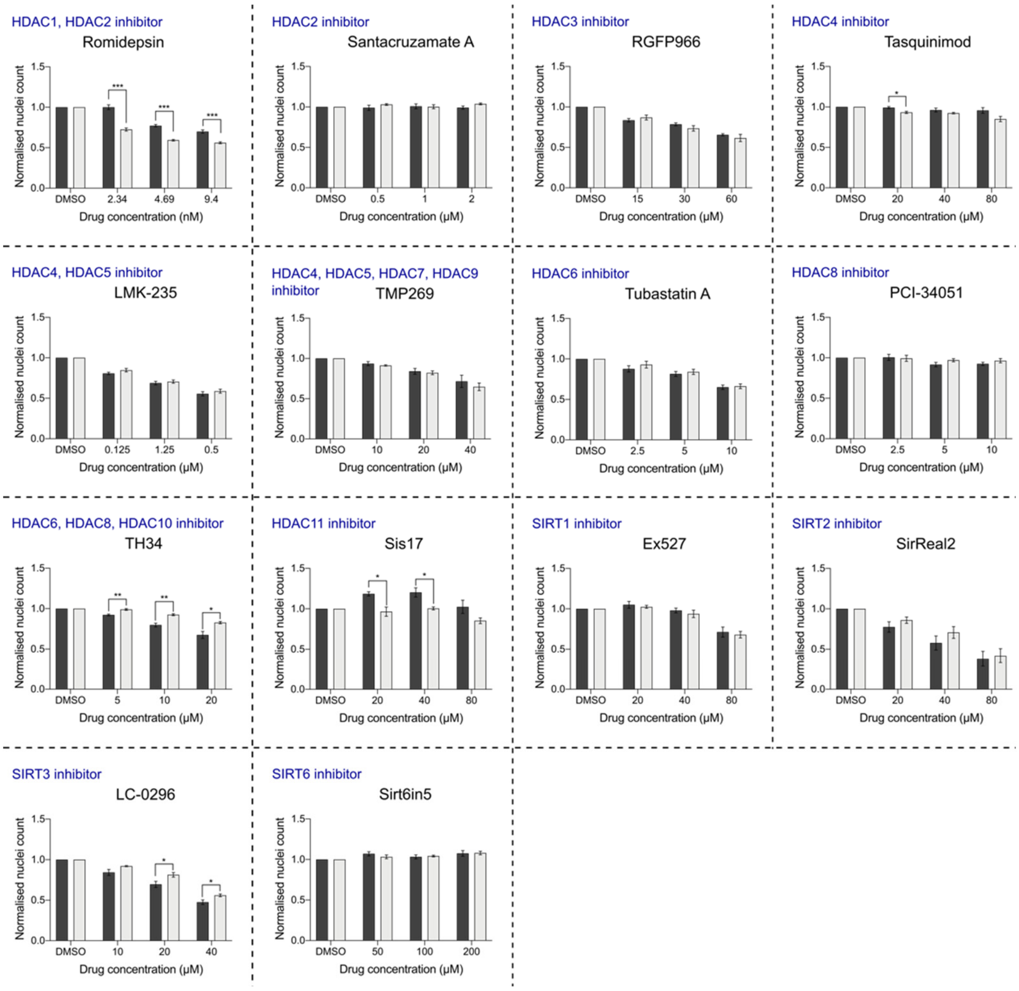

Figure S3. Specific HDAC inhibitors have less effect on NCI-N87 cells compared to pan-HDAC inhibitors. NCI-N87-WT and NCI-N87-*CDH1*$^{-/-}$ cells were treated with specific HDAC inhibitors for 24h (Romidepsin) or 72h (for the other compounds). The fold change of cell nuclei number after each drugging was measured compared to their respective DMSO. A total of 8 concentrations has been tested for each compound following the literature recommendation to inhibit specifically certain HDAC. Of all the compound tested, only Romidepsin (HDAC1 and 2 inhibitors), Tasquinimod (HDAC4 inhibitor) and Sis17 (HDAC11) present a small SL effect. TH34 (HDAC6, 8 and 10 inhibitor) and LC-0296 (SIRT3 inhibitor) induced a reverse synthetic lethal effect. n=4-7 per compound. Statistically significant results are labeled as * $p < 0.05$, ** p < 0.01 and *** $p < 0.001$.

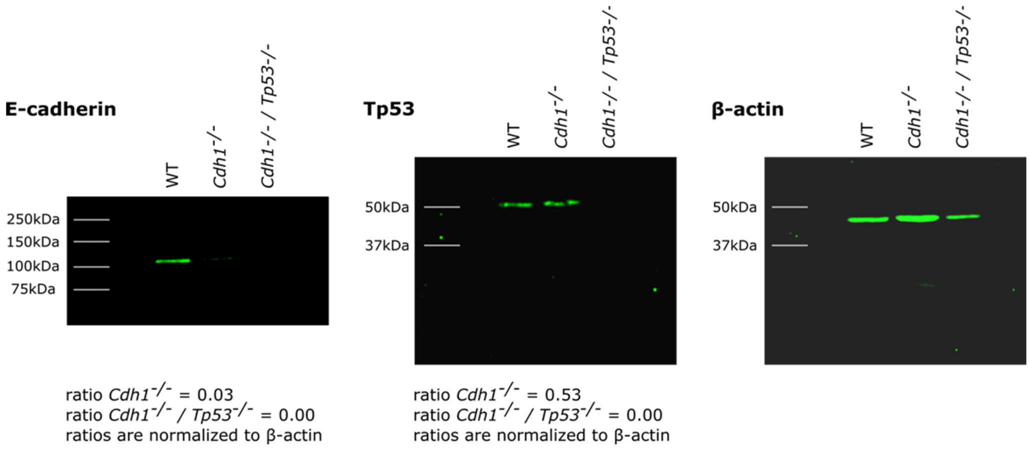

Figure S4. E-cadherin and Tp53 expression in WT and E-cadherin-deficient gastric organoids. Representation of the whole western blot showed in figure 4A with protein extracted from WT, *Cdh1*$^{-/-}$ and *Cdh1*$^{-/-}$ / *Trp53*$^{-/-}$ organoids. E-cadherin staining was observed at 110kDa, P53 at 53kDa and β-actin at 45kDa. The intensity ratios were normalized to β-actin.

Table S1. List of the different compounds used for the drugging experiment with their respective targets.

Drug name	Targets	Reference
Entinostat	Pan-HDAC	Aobious, AOB2570
Pracinostat	Pan-HDAC with exception for HDAC6 and SIRT1	Aobious, AOB87709
Mocetinostat	HDAC1, HDAC2, HDAC3, HDAC11	Aobious, AOB87751
Vorinostat	Pan-HDAC	Aobious, AOB6083
Romidepsin	HDAC1, HDAC2	Aobious, AOB1853
Santacruzamate A	HDAC2	GlixxLab, GLXC-07507
RGFP966	HDAC3	Aobious, AOB6208
Tasquinimod	HDAC4	Aobious, AOB87360
LMK235	HDAC4, HDAC5	GlixxLab, GLXC-03900
TMP269	HDAC4, HDAC5, HDAC7, HDAC9	Aobious, AOB6266
Tubastatin A	HDAC6	Aobious, AOB6340
PCI-34051	HDAC8	GlixxLab, GLXC-90446
TH34	HDAC6, HDAC8, HDAC10	Selleckchem, S8773
SIS17	HDAC11	Selleckchem, S6687
Ex525	SIRT1	Aobious, AOB2079
SirReal2	SIRT2	Aobious, AOB5184
LC-0296	SIRT3	Aobious, AOB37996
Sirt6in5	SIRT6	Aobious, AOB33587

Table S2. Gastric organoid complete media.

Compound	Concentration	Reference
Advanced DMEM/F12	N/A	Life technologies, 12634028
HEPES	10mM	Thermo Fisher, 15630-106
GlutaMAX	2mM	Thermo Fisher 35050061
Pen/Strep	1X	Life technologies, 15140148
N-acetylcysteine	1mM	Sigma, A9165-5G
Gastrin	10nM	Sigma, G9145
hEGF	50ng/ml	Sigma, E9644
R-spondin1_conditioned medium	10%	harvested from HA-R-Spondin1-Fc 293T Cells
Noggin_conditioned medium	10%	harvested from HEK293-mNoggin-Fc cells
hFGF10	100ng/ml	Abacus dx, 100-26
Wnt3a_conditioned medium	50%	harvested from L-Wnt3a cells, ATCC CRL-2647
Y-27632 dihydrochloride	10μM	Sigma, Y0503
B-27 supplement	1X	Thermo Fisher, 17504044
N-2 supplement	1X	Thermo Fisher, 17502048
A83-01	2μM	Sapphire, 9001799

Table S3. Mammary organoid complete media.

Compound	Concentration	Reference
Advanced DMEM/F12	N/A	Life technologies, 12634028
HEPES	10mM	Thermo Fisher, 15630-106
GlutaMAX	2mM	Thermo Fisher 35050061
Pen/Strep	1X	Life technologies, 15140148
N-acetylcysteine	1.25μM	Sigma, A9165-5G
hEGF	50ng/ml	Sigma, E9644
R-spondin1_conditioned medium	1%	harvested from HA-R-Spondin1-Fc 293T Cells
hFGF10	10ng/ml	Abacus dx, 100-26
Wnt3a_conditioned medium	3%	harvested from L-Wnt3a cells, ATCC CRL-2647
Y-27632 dihydrochloride	5μM	Sigma, Y0503
B-27 supplement	1X	Thermo Fisher, 17504044
A83-01	1μM	Sapphire, 9001799
Insulin	5μg/ml	Sigma, I0516
Hydrocortisone	100ng/ml	Sigma, H0888
mFGF2	5ng/ml	Peprotech, 450-33

Table S4. Adjusted *p-value* of the Spearman correlation between HDACs and *CDH1* expression.

ID	Class	Spearman Correlation	*Adj.p-value*
HDAC1	I	0.32	1.03×10^{-6}
HDAC2	I	0.10	3.17×10^{-1}
HDAC3	I	0.16	1.00
HDAC4	IIa	-0.21	1.30×10^{-6}
HDAC5	IIa	-0.23	7.15×10^{-6}
HDAC6	IIb	0.19	4.91×10^{-2}
HDAC7	IIa	-0.27	5.24×10^{-7}
HDAC8	I	0.06	1.00
HDAC9	IIa	-0.20	8.18×10^{-5}
HDAC10	IIb	0.26	3.09×10^{-5}
HDAC11	IV	0.05	1.00
SIRT1	III	-0.12	6.66×10^{-1}
SIRT2	III	0.00	1.00
SIRT3	III	0.03	1.00
SIRT4	III	-0.15	2.39×10^{-2}
SIRT5	III	0.16	1.08×10^{-2}
SIRT6	III	0.12	9.73×10^{-1}
SIRT7	III	0.37	6.73×10^{-13}

Table S5. IC50 of the different pan-HDAC inhibitors obtained on breast and gastric cancer cells and on mammary and gastric organoids.

Drug name	MCF10A	NCI-N87	Gastric organoids	Mammary organoids
Entinostat	WT_2.2µM<br>*CDH1*$^{-/-}$_1.7µM	WT_19.5µM<br>*CDH1*$^{-/-}$_3.7µM	WT_>15µM<br>*Cdh1*$^{-/-}$_11.9µM<br>*Cdh1*$^{-/-}$/*Tp53*$^{-/-}$_15µM	WT_>15µM<br>*Cdh1*$^{-/-}$_>15µM
Pracino-stat	WT_0.4µM<br>*CDH1*$^{-/-}$_0.3µM	WT_2µM<br>*CDH1*$^{-/-}$_1.2µM	WT_>1µM<br>*Cdh1*$^{-/-}$_0.8µM<br>*Cdh1*$^{-/-}$/*Tp53*$^{-/-}$_>1µM	WT_>1µM<br>*Cdh1*$^{-/-}$_>1µM
Mocetino-stat	WT_1.3µM<br>*CDH1*$^{-/-}$_1.1µM	WT_>40µM<br>*CDH1*$^{-/-}$_2.1µM	WT_>2.5µM<br>*Cdh1*$^{-/-}$_1.7µM<br>*Cdh1*$^{-/-}$/*Tp53*$^{-/-}$_2.4µM	WT_>10µM<br>*Cdh1*$^{-/-}$_6.67µM
Vorinostat	WT_2.1µM<br>*CDH1*$^{-/-}$_1.6µM	WT_12.6µM<br>*CDH1*$^{-/-}$_3.2µM	WT_>2.5µM<br>*Cdh1*$^{-/-}$_>2.5µM<br>*Cdh1*$^{-/-}$/*Tp53*$^{-/-}$_>2.5µM	WT_>2.5µM<br>*Cdh1*$^{-/-}$_>2.5µM
